# The functional capacity of plantaricin-producing *Lactobacillus plantarum* SF9C and S-layer-carrying *Lactobacillus brevis* SF9B to withstand gastrointestinal transit

**DOI:** 10.1186/s12934-020-01365-6

**Published:** 2020-05-19

**Authors:** Katarina Butorac, Martina Banić, Jasna Novak, Andreja Leboš Pavunc, Ksenija Uroić, Ksenija Durgo, Nada Oršolić, Marina Kukolj, Slobodanka Radović, Simone Scalabrin, Jurica Žučko, Antonio Starčević, Jagoda Šušković, Blaženka Kos

**Affiliations:** 1 Laboratory for Antibiotic, Enzyme, Probiotic and Starter Culture Technologies, Faculty of Food Technology and Biotechnology, University of Zagreb, Pierottijeva 6, Zagreb, Croatia; 2 Laboratory for Biology and Microbial Genetics, Faculty of Food Technology and Biotechnology, University of Zagreb, Pierottijeva 6, Zagreb, Croatia; 3 Department of Animal Physiology, Faculty of Science, University of Zagreb, Rooseveltov trg 6, Zagreb, Croatia; 4 IGA Technology Services srl, via Jacopo Linussio 51, Udine, Italy; 5 Laboratory for Bioinformatics, Faculty of Food Technology and Biotechnology, University of Zagreb, Pierottijeva 6, Zagreb, Croatia

**Keywords:** Antibacterial activity, Gut colonisation, *Lactobacillus*, Microbiota, Plantaricin, S-layer

## Abstract

**Background:**

We evaluated the functional capacity of plantaricin-producing *Lactobacillus plantarum* SF9C and S-layer-carrying *Lactobacillus brevis* SF9B to withstand gastrointestinal transit and to compete among the gut microbiota in vivo. Considering the probiotic potential of *Lb. brevis* SF9B, this study aims to investigate the antibacterial activity of *Lb. plantarum* SF9C and their potential for in vivo colonisation in rats, which could be the basis for the investigation of their synergistic functionality.

**Results:**

A plantaricin-encoding cluster was identified in *Lb. plantarum* SF9C, a strain which efficiently inhibited the growth of *Listeria monocytogenes* ATCC^®^ 19111™ and *Staphylococcus aureus* 3048. Homology-based three-dimensional (3D) structures of SF9C plantaricins PlnJK and PlnEF were predicted using SWISS-MODEL workspace and the helical wheel representations of the plantaricin peptide helices were generated by HELIQUEST. Contrary to the plantaricin-producing SF9C strain, the S-layer-carrying SF9B strain excluded *Escherichia coli* 3014 and *Salmonella enterica* serovar Typhimurium FP1 from the adhesion to Caco-2 cells. Finally, PCR-DGGE analysis of the V2–V3 regions of the 16S rRNA gene confirmed the transit of the two selected lactobacilli through the gastrointestinal tract (GIT). Microbiome profiling via the Illumina MiSeq platform revealed the prevalence of *Lactobacillus* spp. in the gut microbiota of the *Lactobacillus*-treated rats, even on the 10th day after the *Lactobacillus* application, compared to the microbiota of the healthy and AlCl_3_-exposed rats before *Lactobacillus* treatment.

**Conclusion:**

The combined application of *Lb. plantarum* SF9C and *Lb. brevis* SF9B was able to influence the intestinal microbiota composition in rats, which was reflected in the increased abundance of *Lactobacillus* genus, but also in the altered abundances of other bacterial genera, either in the model of healthy or aberrant gut microbiota of rats. The antibacterial activity and capacity to withstand in GIT conditions contributed to the functional aspects of SF9C and SF9B strains that could be incorporated in the probiotic-containing functional foods with a possibility to positively modulate the gut microbiota composition.
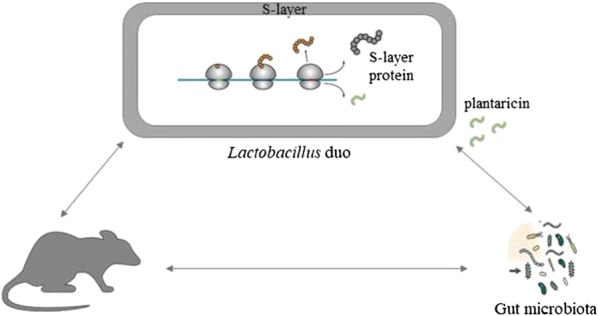

## Background

*Lactobacillus* strains are omnipresent in different ecological niches. The representative members dominate the microbiota of the sauerkraut and are under constant competition with other strains for nutrients and space [13]. The antibacterial activity of *Lactobacillus* strains is an important factor for the pathogen elimination in the complex microbial communities. Bacteriocin-producing *Lactobacillus* strains may achieve a competitive advantage in the surrounding microenvironment, which represents an attractive approach in terms of food biopreservation [13]. Their application expands even to the aspect of health since bacteriocin production is recognised as an important probiotic trait and bacteriocins have even been proposed as alternatives to antibiotics [12, 39]. Bacteriocinogenic activity may contribute to the functionality of probiotics through direct inhibition of the pathogens. Moreover, bacteriocins aid the survival of the producing strain and may act as quorum sensing molecules in the intestinal environment.

Previously, we monitored lactic acid bacteria (LAB) population during spontaneous fermentation of the *Brassica oleracea* var. *capitata* cultivar Varaždinski [6–8]. At the onset of the spontaneous fermentation, LAB diversity was present, including *Leuconostoc mesenteroides* strains, while a restricted number of *Lactobacillus* species, mainly *Lactobacillus plantarum,* dominated in the later stages [7]. *Lb. brevis* SF9B was also isolated from this fermentation. This strain showed desirable functional and technological properties largely influenced by surface (S)-layer proteins (Slps), which were detected by SDS-PAGE [7] and identified by 2D electrophoresis followed by LC–MS analysis. Slps have a functional role in conveying increased survival of the respective SF9B strain under simulated GIT conditions and during freeze-drying. Moreover, the results indicated a prominent role of Slps in adhesion of SF9B strain to mucin, extracellular matrix (ECM) proteins, and particularly to Caco-2 cells [6]. Besides SF9B, at the final stage of spontaneous fermentation, we isolated an autochthonous strain SF9C. This strain was identified as *Lb. plantarum,* which is a prevalent species in sauerkraut fermentation, probably due to its competitiveness with autochthonous microbiota. Turbidimetric method had previously revealed antibacterial activity of *Lb. plantarum* SF9C against some common pathogens [7]. Therefore, the aim of this study is to evaluate the competitive advantage potential of bacteriocin-producing *Lb. plantarum* SF9C and S-layer-carrying *Lb. brevis* SF9B against pathogens by in vitro and in vivo investigations. It also tests possible bacteriocinogenic activity of SF9C strain against Gram-positive pathogens *Listeria monocytogenes* ATCC^®^ 19111™ and *Staphylococcus aureus* 3048. Coculturing of LAB strain with common Gram-positive food pathogens stimulates its bacteriocinogenic activity. The stimulation of bacteriocinogenic activity in SF9C strain was performed by its coculturing with common Gram-positive food pathogens. To observe whether the examined *Lactobacillus* strains have a broader spectrum of antimicrobial activity, antagonistic activity against Gram-negative *Escherichia coli* 3014 and *Salmonella* Typhimurium FP1 was also evaluated. Since preclinical evidence indicates that probiotic *Lactobacillus* strains may positively influence gut microbiota composition in different disorders followed by microbiota disturbance [11, 16], this study also aims to assess colonisation potential and the capacity of SF9C and SF9B strains to affect microbiome alterations in vivo, when applied together, either in healthy or AlCl_3_-exposed rats as a model of disturbed microbiota. PCR-DGGE (polymerase chain reaction-denaturing gradient gel electrophoresis) and the Illumina MiSeq sequencing analysis served to investigate if *Lb. brevis* SF9B and *Lb. plantarum* SF9C have the potential for in vivo colonisation and to influence the microbiota composition in the intestinal tract (IT) of rats.

## Results

### Plantaricin-related genes and whole genome sequencing (WGS) of *Lb. plantarum* SF9C

**Fig. 1:**
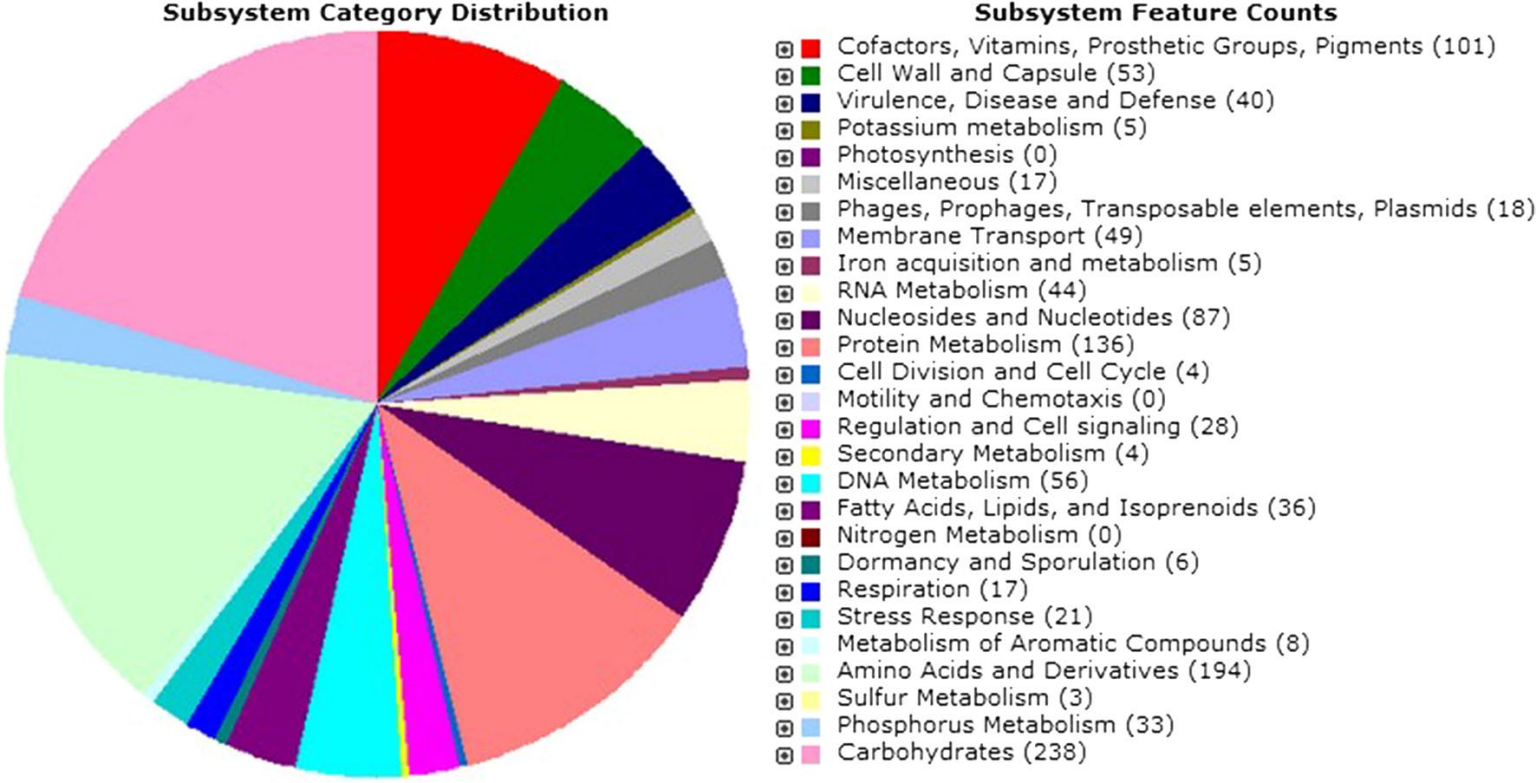
Distribution of *Lb. plantarum* SF9C subsystem gene functions. The complete genome sequence of *Lb. plantarum* SF9C was annotated using the RAST server. The pie chart shows the count of each subsystem feature and the subsystem coverage

PCR gave positive result for plantaricin-related genes *plnA*, *plnE* and *plnJ,* suggesting that SF9C genome could harbour a *pln* locus (Additional file 1: Fig. S1), but it did not detect amplicons for *plnNC8*, *plnS* and *plnW* genes. Rapid Annotations using Subsystems Technology (RAST) of sequences obtained by Illumina MiSeq platform, and tblastn v. 2.2.27 comparison of the assembled contigs with the sequences deposited in NCBI employed for WGS identified SF9C strain as *Lb. plantarum*. This Whole Genome Shotgun project was deposited at DDBJ/ENA/GenBank under the accession RHLZ0000000. The version described in this paper is RHLZ01000000. The genome sequence contained 3.26 million bp (Mb) divided into 14 contigs. The size of *Lb. plantarum* SF9C genome of 3.2 Mb was similar to that of the other members of the species. The number of coding sequences was 3229 and the number of RNAs was 68. According to the comparative genomic studies, the estimated number of predicted protein-coding genes in *Lactobacillus* strains ranges from 1700 to around 3000 [47]. The G + C content of the *Lb. plantarum* SF9C genome was 44.4%, which is similar to the other *Lb. plantarum* strains, e.g. *Lb. plantarum* WCFS1 (44.5%) and *Lb. plantarum* ATCC 14917 (44.5%) [3]. Figure 1 shows the subsystem category distribution of major protein encoding genes (PEGs) for *Lb. plantarum* SF9C annotated by RAST server. The pie chart depicts the percentage distribution of 27 most abundant subsystem categories in SF9C strain. While most of the PEGs were related to universal cell functions such as DNA replication, transcription, translation, ribosomal structure and biogenesis, protein turnover and chaperones, and transport and metabolism of carbohydrates and nucleotides, certain PEGs were associated with the specific categories of cellular defence mechanisms and secondary metabolite biosynthesis, transport and catabolism, which may be responsible for the antimicrobial phenotype of SF9C strain.

**Fig. 2:**
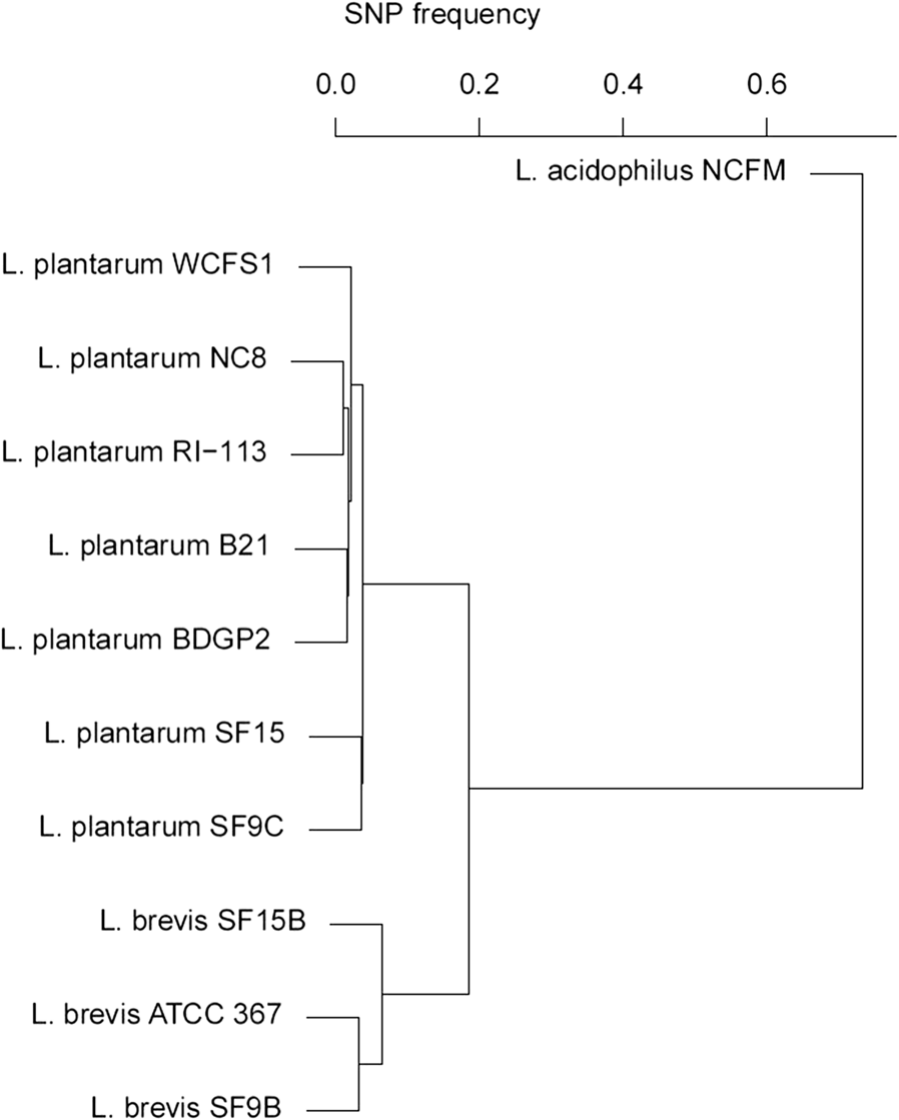
Hierarchical clustering of multiple *Lactobacillus* genomes based on single-nucleotide polymorphism (SNP) frequency. SNP frequency is “number of bases divided by bases aligned”. Origins and resources of the respected strains: WCFS1 [29], NC8 [4], RI-113 [28], B21 [23], BDGP2 [49], SF15C, SF9C, SF15B and SF9B [8], ATCC367 [36] and NCFM [1]

Given that SF9B and SF9C originate from the same microenvironment, their whole genomes were compared and the cluster dendrogram that reflects the diversity among strains was constructed. Single-nucleotide polymorphism (SNP) hierarchical clustering based on a similarity of whole genome sequences revealed that S-layer-carrying *Lb. brevis* SF9B is grouped with another S-layer-expressing *Lb. brevis* ATCC 367 (Fig. 2). Given that the phylogenetic distance between the strains was small, sauerkraut isolate *Lb. plantarum* SF9C was grouped with SF15C strain, another *Lb. plantarum* strain isolated from the same fermentation batch (Fig. 2).

**Fig. 3:**
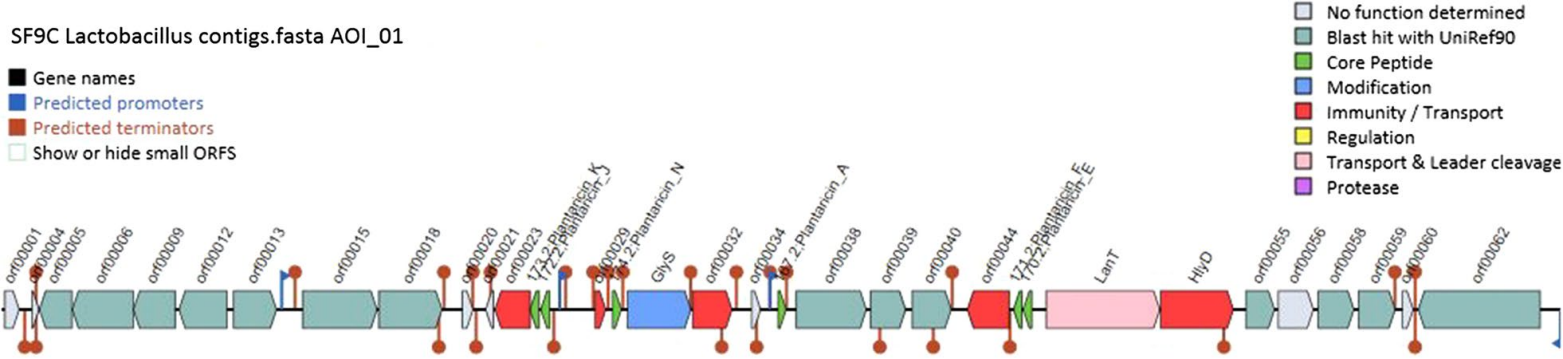
Genetic map of the plantaricin gene cluster of *Lb. plantarum* SF9C strain

**Fig. 4:**
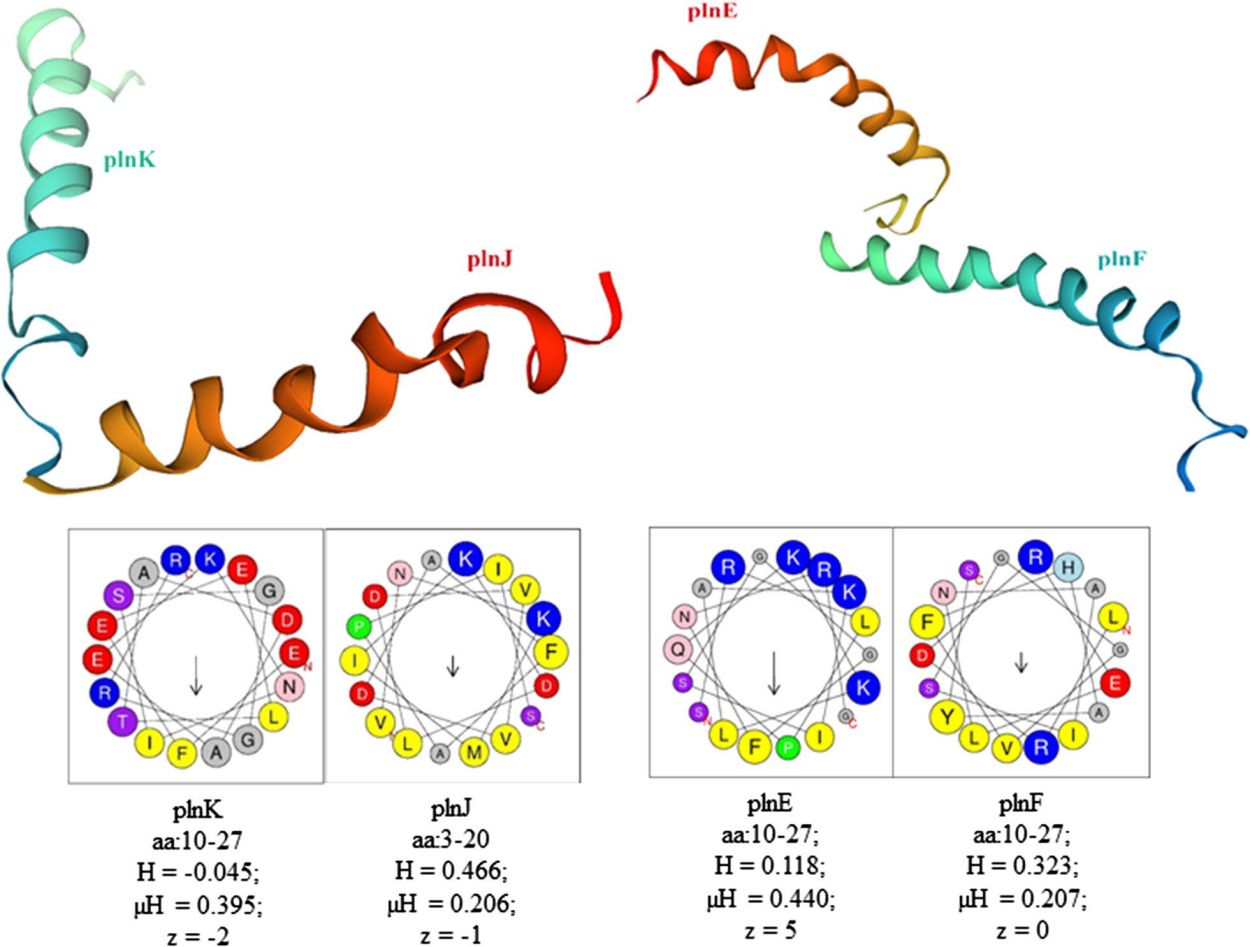
3D structures of PlnJK and PlnEF plantaricin peptides of SF9C strain predicted by the homology modelling and their helical wheel projections analysed by HeliQuest. aa—the amino acid residues (the one-letter code for amino acids is used); yellow—hydrophobic residues; purple—serine and threonine residues; dark blue—basic residues; red—acidic residues, pink—asparagine and glutamine residues; grey—alanine and glycine residues, light blue—histidine residues; green—proline residues; H—hydrophobicity; μH—hydrophobic moment; z—net charge (calculated at pH = 7.4, under the assumption that histidine is neutral and that the N-terminal amino group and the C-terminal carboxyl group of the sequence are uncharged)

Next, WGS data were exploited to identify potential genomic triggers that may be responsible for the antibacterial phenotype. The assembled contigs were compared with the bacteriocins identified so far in the NCBI database using the tblastn v. 2.2.27. Through functional annotation and analysis of the high-coverage contigs obtained through Illumina sequencing, the genes involved in plantaricin production were predicted for *Lb. plantarum* SF9C and compared with the genes of other *Lb. plantarum*-bacteriocin-producing strains (Additional file 2: Table S1). The genome sequence of *Lb. plantarum* SF9C contains a cluster for biosynthesis of a putative plantaricin. In silico BAGEL4 analysis identified one area of interest (AOI) located at contig 13. The *pln* locus of SF9C contains genes encoding their cognate immunity proteins, whose location is just downstream of the bacteriocin genes, as well as ABC transporters, probably involved in the export of peptides with a double glycine leader (Fig. 3). Finally, the SWISS-MODEL predicted homology-based three-dimensional (3D) structures of SF9C two-peptide plantaricins PlnJK and PlnEF. Properties of the chosen amino acid (aa) residues that form a helix of each of the two plantaricins, PlnJK and PlnEF, were calculated by HeliQuest web server (Fig. 4).

### Antimicrobial activity of *Lb. plantarum* SF9C after the cocultivation with pathogens

Preliminary results regarding the antagonistic activity of the two naturally coexisting strains *Lb. plantarum* SF9C and *Lb. brevis* SF9B, clearly demonstrated the difference in the spectrum of antibacterial activity (Tables 1, 2). Grown cultures of both strains, SF9C and SF9B, demonstrated antibacterial activity against *L. monocytogenes* ATCC^®^ 19111™, *S. aureus* 3048, *E. coli* 3014 and *S.* Typhimurium FP1 (Tables 1, 2). Cell-free supernatant (CFS) of the strain SF9C exerted the antibacterial activity against the selected pathogens, while CFS of the strain SF9B did not inhibit the examined pathogens (Table 2). Furthermore, the combined CFSs of both strains, SF9C and SF9B, in equal ratio, showed decreased antibacterial activity against *L. monocytogenes* ATCC^®^ 19111™, *S. aureus* 3048, *E. coli* 3014 and *S*. Typhimurium FP1, which was expected since the CFS of S-layer-carrying SF9B strain did not demonstrate antibacterial activity against these pathogens, and therefore alleviated cumulative inhibitory effect of combined CFSs (Table 2).

**Table 1:** Inhibition of *Listeria monocytogenes* ATCC^*®*^ 19111™, *Staphylococcus aureus* 3048, *Escherichia coli* 3014 and *Salmonella* Typhimurium FP1 by grown cultures of *Lb. plantarum* SF9C and *Lb. brevis* SF9B, separately and combined, evaluated by agar spot-test method and expressed as the diameter of inhibition zones around the grown cultures (cm)

*Lactobacillus* strains	Diameter of inhibition zone (cm)			
	*L. monocytogenes* ATCC^*®*^ 19111™	*S. aureus* 3048	*E. coli* 3014	*S.* Typhimurium FP1
*Lb. plantarum* SF9C	3.50 ± 0.10^az^	3.32 ± 0.13^azx^	3.05 ± 0.05^ay^	3.07 ± 0.06^ayx^
*Lb. brevis* SF9B	1.93 ± 0.40^bz^	1.35 ± 0.05^by^	1.75 ± 0.31^bzy^	1.52 ± 0.08^bzy^
Combined SF9C + SF9B	2.57 ± 0.06^bz^	1.50 ± 0.10^bx^	2.17 ± 0.06^by^	2.60 ± 0.10^cz^

^abc^Different letter means statistically significant difference (*p *< 0.05) within the same column among the used *Lactobacillus* strains

^xyz^Different letter means statistically significant difference (*p *< 0.05) within the same row among the used pathogens. Statistical analysis was carried out using ANOVA and the results are reported as mean value ± SD of three independent experiments

**Table 2:** Comparison of the antimicrobial activity of cell-free supernatants (CFSs) of *Lb. plantarum* SF9C and *Lb. brevis* SF9B, separately and combined, before and after the treatment with proteinase K and high temperature heating, against *L. monocytogenes* ATCC^®^ 19111™, *S. aureus* 3048, *E. coli* 3014 and *S.* Typhimurium FP1, determined by agar well-diffusion assay and expressed as the diameter of inhibition zones around the wells (cm)

Treatment of CFS	*Lactobacillus* strains	Diameter of inhibition zone (cm)			
		*L. monocytogenes* ATCC^®^ 19111™	*S. aureus* 3048	*E. coli* 3014	*S.* Typhimurium FP1
Before treatment	*Lb. plantarum* SF9C	1.92 ± 0.03^az^	1.57 ± 0.06^ay^	1.50 ± 0.05^ayw^	1.38 ± 0.03^aw^
	*Lb. brevis* SF9B	0.00 ± 0.00^ez^	0.00 ± 0.00^ez^	0.00 ± 0.00^ez^	0.00 ± 0.00^fz^
	Combined SF9C + SF9B	1.72 ± 0.03^bz^	0.98 ± 0.03^dx^	1.13 ± 0.06^cy^	1.00 ± 0.00^cx^
proteinase K	*Lb. plantarum* SF9C	1.10 ± 0.00^dy^	1.17 ± 0.03^cyz^	1.23 ± 0.03^bz^	1.20 ± 0.00^bxz^
	*Lb. brevis* SF9B	0.00 ± 0.00^ez^	0.00 ± 0.00^ez^	0.00 ± 0.00^ez^	0.00 ± 0.00^fz^
	Combined SF9C + SF9B	1.02 ± 0.03^dz^	0.90 ± 0.00^dy^	0.98 ± 0.03^dz^	0.85 ± 0.00^ey^
100 °C/30 min	*Lb. plantarum* SF9C	1.68 ± 0.03^bz^	1.52 ± 0.03^by^	1.30 ± 0.00^bx^	1.23 ± 0.03^bx^
	*Lb. brevis* SF9B	0.00 ± 0.00^ez^	0.00 ± 0.00^ez^	0.00 ± 0.00^ez^	0.00 ± 0.00^fz^
	Combined SF9C + SF9B	1.53 ± 0.06^cz^	0.93 ± 0.06^dx^	1.03 ± 0.06^cdy^	0.92 ± 0.03^dx^

^abcdef^Different letter means statistically significant difference (*p *< 0.05) within the same column among the treatments of CFSs and used *Lactobacillus* strains

^wxyz^Different letter means statistically significant difference (*p *< 0.05) within the same row among the used pathogens. Statistical analysis was carried out using ANOVA and the results are reported as mean value ± SD of three independent experiments

*Lb. plantarum* SF9C strain showed the strongest antibacterial activity against the closely related Gram-positive pathogen *L. monocytogenes* ATCC^®^ 19111™, implying the potential bacteriocinogenic activity (Tables 1, 2). Therefore, CFSs of both examined *Lactobacillus* strains, separately and combined, were treated with proteinase K and exposed to high temperature in order to inactivate the potentially present bacteriocin. According to the obtained results, the antibacterial activity of the CFS of SF9C strain as well as combined CFSs of both *Lactobacillus* strains was partially inactivated after the treatment with proteinase K and after its exposure to high temperature of 100 °C for 30 min, compared to the CFSs that were not treated with proteinase K or high temperature (Table 2). These findings confirmed the presence of a substance with a proteinaceous nature in CFS of *Lb. plantarum* SF9C.

Similarly, the evaluation of the antibacterial activity of SF9C and SF9B against closely related LAB strains by agar well-diffusion method showed that SF9C was more effective in the inhibition of the examined LAB strains than SF9B, with the strongest effect observed against *Enterococcus*, moderate against *Lactococcus* and the weakest against *Lactobacillus* strains (data not shown).

**Fig. 5:**
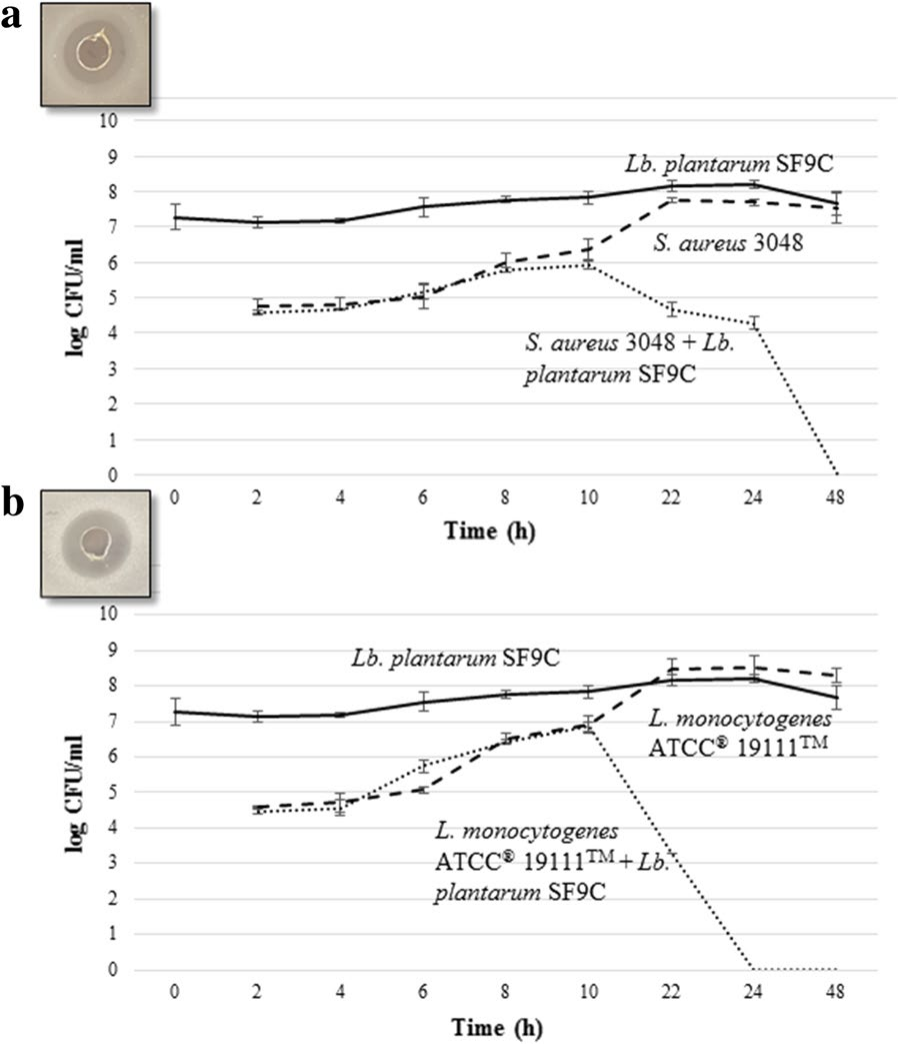
The growth curves of the test microorganisms: a) *S. aureus* 3048 and b) *L. monocytogenes* ATCC^®^ 19111™ during cocultivation with (···) or without (▬ ▬) *Lb. plantarum* SF9C. Growth curve of *Lb. plantarum* SF9C (▬)

To assess the possibility to enhance the bacteriocin activity of *Lb. plantarum* SF9C, this strain was cocultivated with *S. aureus* 3048 and *L. monocytogenes* ATCC^®^ 19111™, since the activity of bacteriocins from LAB is mostly directed towards Gram-positive bacteria. The growth of *Lb. plantarum* SF9C strain was not impaired by the cocultivation with Gram-positive pathogens, but the log CFU/mL values of *S. aureus* 3048 and *L. monocytogenes* ATCC^®^ 19111™ were reduced to non-detectable levels after 48 and 24 h of cocultivation, respectively (Fig. 5). Additionally, the antibacterial effect of *Lb. plantarum* SF9C, obtained by agar spot test, was significantly higher after 8, 10 and 22 h of incubation in coculture with *S. aureus* 3048, as well as after 22, 24 and 48 h of incubation in coculture with *L. monocytogenes* ATCC^®^ 19111™ (Additional file 3: Fig. S2). The obtained results indicate the possibility to enhance antibacterial activity of *Lb. plantarum* SF9C by incubation in the presence of the sensitive Gram-positive pathogens.

### Inhibition of pathogen adherence to Caco-2 cells by *Lb. brevis* SF9B

Competitive pathogen exclusion assays by *Lb. plantarum* SF9C and *Lb. brevis* SF9B on Caco-2 human intestinal cells were performed. Since *Lb. plantarum* SF9C strain exhibited stronger antimicrobial activity against Gram-positive than against Gram-negative pathogens, as revealed by agar spot-test and agar well-diffusion assays, the possibility of SF9C to exclude targeted *L. monocytogenes* ATCC^®^ 19111™ and *S. aureus* 3048 from the Caco-2 cell line was investigated. After 1 h of incubation, the number of adhered pathogens exposed to *Lb. plantarum* SF9C strain reached the values of (5.916 ± 0.527) log CFU/mL of *L. monocytogenes* ATCC^®^ 19111™ and (7.167 ± 0.168) log CFU/mL of *S. aureus* 3048, respectively, which was not significantly reduced (*p *< 0.05) compared to the control ((5.823 ± 0.204) log CFU/mL of *L. monocytogenes* ATCC^®^ 19111™ and (7.448 ± 0.181) log CFU/mL of *S. aureus* 3048, respectively).

While SF9C strain failed in preventing Caco-2 adhesion of the tested pathogens, *Lb. brevis* SF9B significantly reduced the adhesion of Gram-negative pathogens *S.* Typhimurium FP1 (*p *< 0.05) and *E. coli* 3014 (*p *< 0.01) in competitive exclusion assays. Namely, in exclusion assay, when Caco-2 cells were exposed to *Lb. brevis* SF9B before *S.* Typhimurium FP1, significantly fewer (*p *< 0.01) *Salmonella* cells adhered to them ((4.708 ± 0.014) log CFU/mL) than after exposure of Caco-2 cells to *S.* Typhimurium FP1 alone ((6.825 ± 0.099) log CFU/mL). In competition assay, when Caco-2 cells were incubated simultaneously with *Lb. brevis* SF9B and *Salmonella,* significantly fewer (*p *< 0.05) *Salmonella* cells adhered to them ((5.613 ± 0.135) log CFU/mL) than to Caco-2 cells infected with *Salmonella* alone ((6.825 ± 0.099) log CFU/mL). The competitive exclusion effect of *Lb. brevis* SF9B was even stronger against *E. coli* 3014, where the significant inhibition (*p *< 0.01) of an invasion of Caco-2 cells by *E. coli* 3014 in comparison to the control (without the addition of SF9B cells) was evident, with reduced values of 2.209 Δlog CFU/mL when pathogen cells were added after SF9B cells (exclusion), and of 2.117 Δlog CFU/mL when *Lb. brevis* SF9B and pathogen cells were added simultaneously (competition).

### Influence of *Lb. brevis* SF9B and *Lb. plantarum* SF9C on gut microbiome composition

**Fig. 6:**
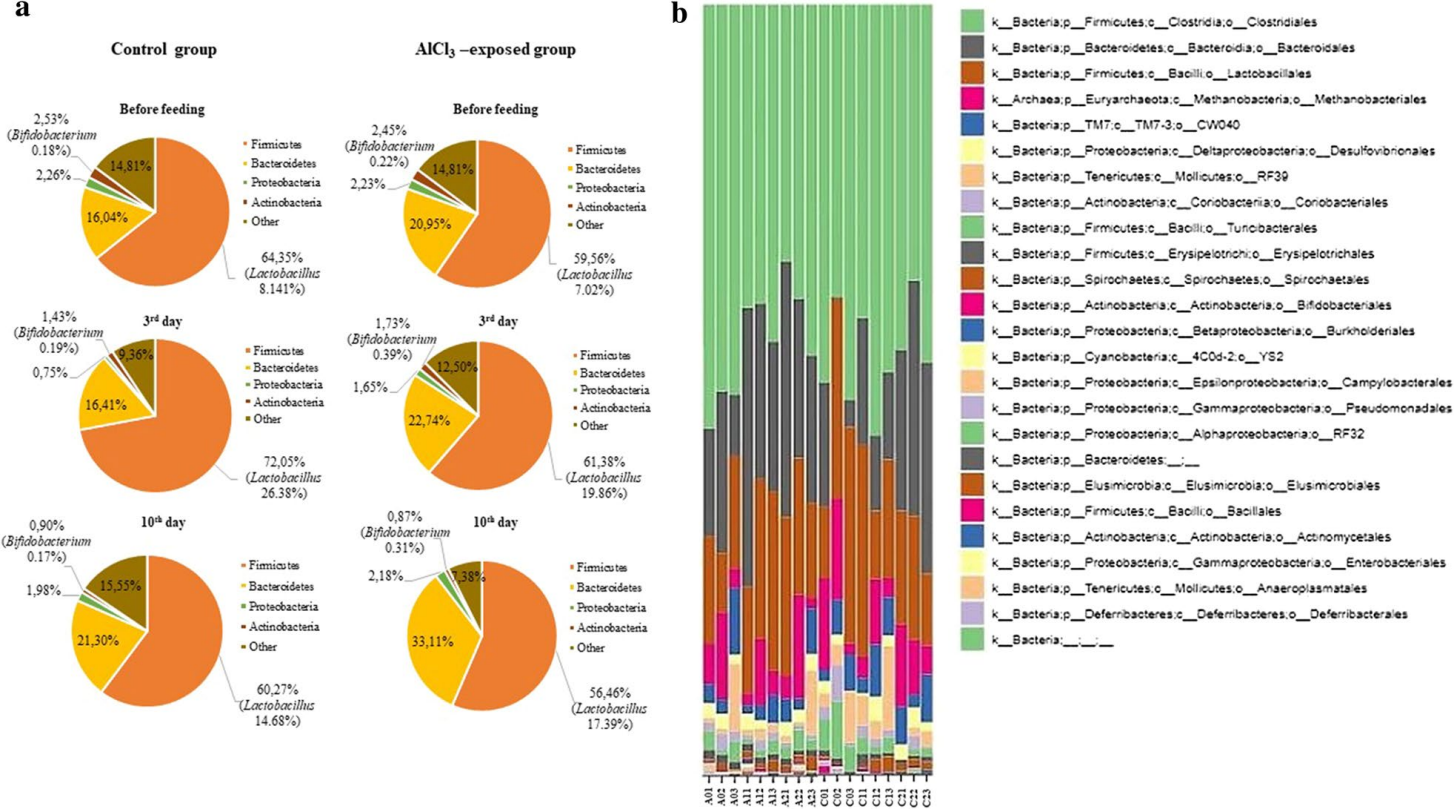
**a** The four most abundant phyla detected in the faecal microbiota of control and AlCl_3_-exposed rats, both fed with *Lb. plantarum* SF9C and *Lb. brevis* SF9B. **b** The distribution of the bacterial classes in the faeces of control (C) and AlCl_3_-exposed rats (A), before application (0), and on the 3rd day (1), and 10th day (2) after the application of SF9B and SF9C strains. The second number represents the ordinal number of the rat

**Fig. 7:**
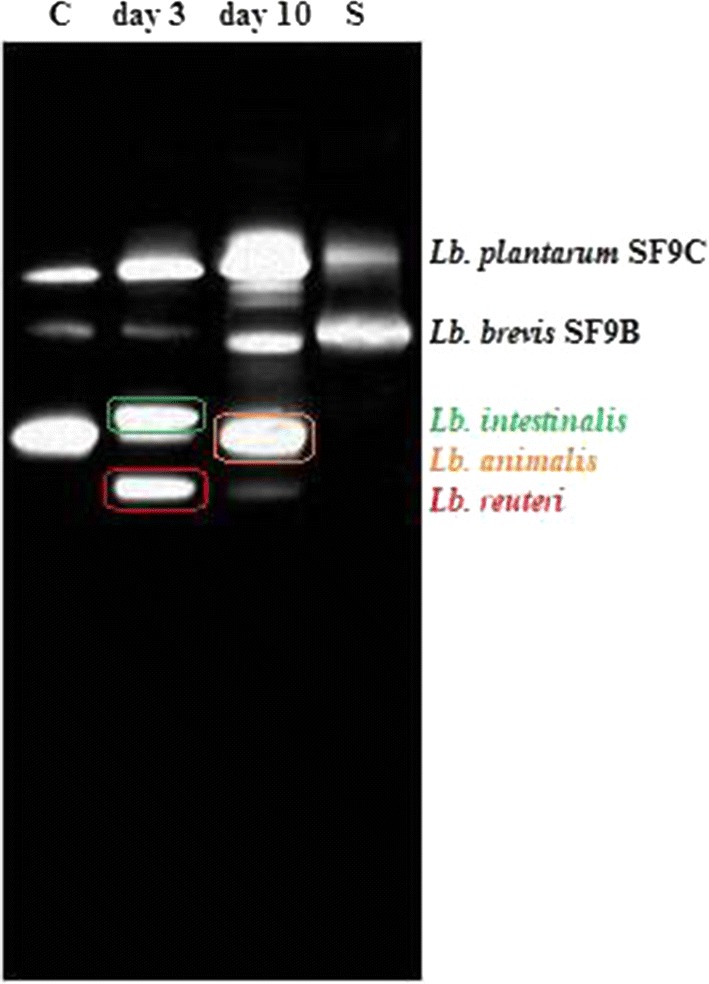
PCR-DGGE analysis of 16S DNA fragments generated with the universal bacterial primers HDA1 and HDA2 from the pooled DNA samples of the *Lactobacillus* species, isolated on MRS agar from faecal samples of rats fed with *Lb. plantarum* SF9C and *Lb. brevis* SF9B. Lanes: C—before application of SF9C and SF9B strains; day 3—3rd day after the application of SF9C and SF9B strains; day 10—10th day after application of SF9C and SF9B strains, S—the ladder of sequences from the pure cultures of SF9C and SF9B strains, respectively. Bands indicated by the symbols were excised and after amplification sequenced

To assess the capacity of the two *Lactobacillus* strains to survive transit and eventually colonize the GIT, the gut microbiome composition after their transit through the GIT was monitored in vivo. Since the aberrant microbiota differs from the microbiota of healthy subjects in the prevalence of undesirable species, the AlCl_3_-exposed rats were chosen as an animal model for microbiome dysbiosis because previous research had shown that toxic metals like aluminium had a negative impact directly on the gut microbiota in humans and animals. Additionally, *Lactobacillaceae*, particularly *Lb. plantarum*, *Lb. rhamnosus* and *Lb. brevis*, were able to bind and remove toxic metals [50]. A recent study by Tian et al. [43] also suggests the potential of the *Lb. plantarum* strain to alleviate the aluminium-induced brain injuries in mice. Therefore, the potential of *Lb. plantarum* SF9C and *Lb. brevis* SF9B to compete among microbiota of AlCl_3_-exposed rats was investigated. The acetylcholinesterase (AChE) activity was assessed in the brain tissue homogenates to monitor the possible influence of AlCl_3_ treatment in the rats. The AChE activity and histopathological and immunohistochemical analyses of the brain showed that the number of plaques and the AChE activity were significantly higher in the brains of the AlCl_3_-exposed group than in the control group (*p *< 0.05) [34]. According to the obtained results, the neuropathological changes were observed in the AlCl_3_-exposed rats. Diffuse plaques, also called benign plaques, occurred much earlier than the neuritic plaques in the cerebellum. In the treatments, cerebellum of the AlCl_3_-exposed rats was negative on the AT8 marker, but positive on 4G8 and Iba1 markers (Additional file 4: Fig. S3). The value of AChE in the control group was lower (from 1.2 to 1.58 mol of hydrolyzed substrate/min/mg protein) than of the AlCl_3_-exposed group (from 1.38 to 2.4 mol of hydrolyzed substrate/min/mg protein). Furthermore, the analysis of faecal microbiota of rats by Illumina MiSeq sequencing revealed that across all, control or AlCl_3_-exposed groups (calculated as mean values of all the experiments), the dominant phyla were *Firmicutes* and *Bacteroidetes*, which respectively made up 63% (62.35 ± 5.40%) and 22% (21.76 ± 6.20%) of total abundance, with lower contributions from *Actinobacteria* (1.65 ± 0.73%) and *Proteobacteria* (1.84 ± 0.58%) (Fig. 6a). The *Firmicutes* and *Bacteroidetes* phyla accounted for more than 85% of total sequences, similar to previous findings in the gut microbiota of rats. However, phylum- through genus-wide differences in bacterial abundance were observed between these two groups. In the microbiome of AlCl_3_-exposed rats the abundance of *Firmicutes* and *Actinobacteria* decreased, while the abundance of *Bacteroidetes* increased compared to the control group. At the class level, the most abundant in all groups were *Bacilli*, *Clostridia* and *Bacteroidia* (Fig. 6b). *Bifidobacterium* was also consistently detected through the samples (Fig. 6a). Since our main goal was to evaluate the survival and colonisation potential of the two *Lactobacillus* strains in the model of healthy, but also in animals with disturbed microbiota, the focus was on the evaluation of *Lactobacillus* abundance. The abundance in *Lactobacillus* sp. was observed in all treated rat groups, implying the ability of SF9B and SF9C to adapt to the GIT, especially since these two strains are not of an intestinal, but sauerkraut origin. The gut microbiome analysis revealed taxonomic differences in gut microbiota composition influenced by *Lactobacillus* treatments. The culture-independent PCR-DGGE verified the presence of lactobacilli in the gut microbiota of faecal samples among rats before and after the treatment with SF9B and SF9C strains (Fig. 7). The cultivation on selective agar plates revealed the presence of presumptive *Lactobacillus* in the faeces of the control group at 5.6 × 10^7^ CFU/mL and AlCl_3_-exposed rats at 1.99 × 10^8^ CFU/mL on the 10th day after *Lactobacillus* treatment, which was in correlation with the results of microbiome analysis obtained by the Illumina MiSeq sequencing. PCR-DGGE analysis verified which *Lactobacillus* strains were potentially responsible for the observed higher *Lactobacillus* spp. abundance levels in the microbiota of *Lactobacillus*-treated rats. The PCR-DGGE of DNA fragments obtained by PCR amplification of the V2–V3 regions of the 16S rRNA gene suggested the presence of both *Lactobacillus* strains in the faeces of treated rats since their DNA fragment coincided with the 16S DNA fragment generated from the pure culture of *Lb. brevis* SF9B and *Lb. plantarum* SF9C (Fig. 7). The inoculation of the healthy rats with *Lactobacillus* strains led to the appearance of a new 16S DNA fragment in the PCR-DGGE profile of the sample on the 3rd day after *Lactobacillus* treatment that corresponded to *Lactobacillus reuteri*. Interestingly, the results of microbiota analysis at the species level showed the presence of *Lb. reuteri* and *Lb. brevis* as well. Furthermore, in a PCR-DGGE profile of the healthy rat, an intensive band was consistently detected, assigned after the sequencing and BLAST search to *Lb. animalis*, while on the 3rd day after *Lactobacillus* treatment, a faint band appeared corresponding to *Lactobacillus intestinalis* strain (Fig. 7).

## Discussion

Functional genomics in probiotic research has facilitated the characterisation of candidate *Lactobacillus* strains. Bacteriocin production is a desirable trait of probiotic strains [25]. Herein, *Lb. plantarum* SF9C genome sequence was determined using a WGS assembly approach, with a focus on the characterisation of the plantaricin locus. The WGS confirmed the presence of the plantaricin (*pln*) loci in SF9C strain. The *plnE* and *plnF* genes that encode for bacteriocin precursor peptide, the *plnA* which encodes induction factor and individual gene *plnJ* were also detected by PCR (Additional file 1: Fig. S1). Plantaricin EF (PlnEF) and plantaricin JK (PlnJK) have already been described in certain *Lb. plantarum* strains as two-peptide bacteriocins. These compounds are biosynthesised as prepeptides and are cleaved off during the transport to the cell surface to become active peptides whose activity depends on the complementary action of the two peptides PlnE/PlnF, i.e. PlnJ/PlnK [15]. Predicted 3D structures of two *Lb. plantarum* SF9C plantaricins by SWISS homology modelling showed sequence similarity with the structures of PlnJK and PlnEF described by Rogne et al. [41] and Fimland et al. [19], respectively.

Bacteriocin activity of the particular strain together with its ability to compete for limited nutrients, competitive exclusion, and the stimulation of mucosal immunity could contribute to intestinal health [17]. Herein the antibacterial activity of the *Lb. plantarum* SF9C against Gram-positive *L. monocytogenes* ATCC^®^ 19111™ and *S. aureus* 3048, and Gram-negative pathogens *E. coli* 3014 and *S.* Typhimurium FP1 was determined. *Lb. plantarum* SF9C drastically decreased the pH value (3.86 ± 0.04) after overnight growth due to the lactic acid production (2.25 ± 0.24% v/v), which creates unfavourable microenvironment for the pathogenic bacteria. The mechanisms of the antibacterial activity of SF9C strain are multifactorial and include the inhibition by the produced lactic acid, but also the potential antibacterial activity of plantaricin, especially since the inhibition was alleviated after the treatment with proteinase K and boiling of CFS of *Lb. plantarum* SF9C. *L. monocytogenes* ATCC^®^ 19111™ and *S. aureus* 3048 possess several mechanisms to combat the challenges posed by acidic environments and therefore can tolerate low pH values. This claim was supported by the finding that *Lb. plantarum* SF9C strain demonstrated antibacterial activity against *L. monocytogenes* ATCC^®^ 19111™ and *S. aureus* 3048, unlike *Lb. brevis* SF9B, which failed to inhibit respective pathogens, even though it is an effective lactic acid producer. Additionally, *L. monocytogenes* and *S. aureus* were deliberately chosen since these foodborne Gram-positive pathogens contaminate a wide range of fermented foods, although the pH value in these food matrices is low due to the metabolic activity of a spontaneously present population of LAB. Therefore, it was hypothesized that the potential plantaricin antibacterial activity was involved in the growth inhibition of Gram-positive pathogens, *L. monocytogenes* and *S. aureus.* Since one strategy to achieve the expression of otherwise silenced bacteriocins is the stimulation of their biosynthesis by growth in the coculture [10, 30, 38], the potential to enhance plantaricin antibacterial activity by cocultivation of *Lb. plantarum* SF9C with *S. aureus* 3048 and *L. monocytogenes* ATCC^®^ 19111™ was studied. Antibacterial activity was initially detected in the early exponential phase of the pathogen growth after 10 h of incubation. The highest antibacterial activity was observed after 24 h in the late exponential phase of *L. monocytogenes* ATCC^®^ 19111™ and after 48 h of *S. aureus* 3048. This is supported by the results of Maldonado-Barragán et al. [38], who suggested that the induction of bacteriocin production by means of coculturing with specific bacterial strains is a common feature among *Lb. plantarum* species. Since *L. monocytogenes* tolerates a broad pH range, it can be speculated that the obtained enhanced antilisterial effect of SF9C during cocultivation with *L. monocytogenes* ATCC^®^ 19111™ is attributed to the potential enhanced plantaricin production. This is in agreement with the feature of *Lactobacillus* bacteriocins whose activity is mostly related towards Gram-positive bacteria.

Contrary to *Lb. plantarum* SF9C, S-layer-carrying *Lb. brevis* SF9B showed competitive exclusion of pathogens on the Caco-2 cells. Slps may act as mediators of bacterial adhesion and as such may contribute to the antagonism against the pathogens with which the S-layer-carrying strain competes for the same adhesion sites [27, 42, 45]. In our previous paper, SF9B strain exhibited the strongest coaggregation with *E. coli* 3014 and *S.* Typhimurium FP1 and the removal of Slps negatively affected its coaggregation ability. This study revealed that S-layer-carrying SF9B strain demonstrated significant levels of exclusion (*p *< 0.01) and competitive capacity (*p *< 0.05) against *S.* Typhimurium FP1, but was more effective in both competitive exclusion experiments against *E. coli* 3014 (*p *< 0.01). Nevertheless, *Lb. brevis* SF9B strain efficiently prevented the adhesion of *S.* Typhimurium FP1 under in vivo conditions [6], probably owing to the considerable coaggregation capacity, as well as longer in vivo coincubation period than the 1 h of coincubation tested in the respective experiment. The coaggregation enables lactobacilli to manipulate a microenvironment around the pathogenic bacteria and inhibit their growth in the gut by secreting antimicrobial substances at their very close proximity. The results suggest that *Lb. brevis* SF9B competed more efficiently with *E. coli* 3014 than *S.* Typhimurium FP1 since the mechanisms of competition and exclusion differ and are highly specific for each pathogen. Combining plantaricin-producing *Lb. plantarum* SF9C with S-layer-carrying *Lb. brevis* SF9B offers an effective strategy to suppress undesirable bacteria such as *L. monocytogenes* ATCC^®^ 19111™, *S. aureus* 3048, *E. coli* 3014 and *S.* Typhimurium FP1 since the joint application of these potential probiotic strains could result in a broader spectrum of antibacterial activity. Furthermore, in contrast to the application of the sole purified bacteriocin, which could be degraded in the GIT, the application of bacteriocin-producer strain assures its continual production and persistence in the gut [24]. Here we suggested the application of the plantaricin-producing *Lb. plantarum* SF9C synergistically with *Lb. brevis* SF9B to eliminate common pathogens, having in mind that *Lb. brevis* SF9B, as a non-bacteriocin producing strain, was found to possess *plnI* gene which encodes for the bacteriocin immunity protein [6]. Both strains, SF9B and SF9C, have shown tolerance of the harsh conditions of the GIT because their relative survival rate decreased only by 2- and 3-log CFU/mL, respectively, under the conditions mimicking the GIT [6, 7]. The cooperation of coexisting *Lactobacillus* strains can also be exploited to control bacterial infection for the reestablishment of the disturbed gut microbiota associated with certain diseases [14]. Therefore, the potential of plantaricin-producing SF9C and S-layer-carrying SF9B strains to compete within healthy or disturbed gut microbiota was examined after their application to the healthy and AlCl_3_-exposed rats. The exposure to AlCl_3_ can cause a variety of adverse physiological effects in humans and animals, including the disturbance of gut microbiota [11, 34]. After *Lactobacillus* treatment of rats, the changes in intestinal microbiota composition were evident, not only in the abundance of *Lactobacillus* genus, but also in the abundance of other bacterial genera. According to the microbiome analysis, *Blautia* genus was not detected in healthy rats but was identified in the AlCl_3_-exposed rats in which its ratio decreased on the 3rd and 10th day after the *Lactobacillus* application. The ratio of *Bacteroides* and *Phascolarctobacterium* genera before the *Lactobacillus* treatment was higher in the AlCl_3_-exposed rats than in the healthy rats, but on the 3rd day after the *Lactobacillus* administration the ratio of these genera was reduced in AlCl_3_-exposed rats while it increased in healthy rats, compared to the ratio of these genera before *Lactobacillus* application. Furthermore, the abundance of the *Bifidobacterium* genus remained unchanged before and after the *Lactobacillus* treatment in both healthy and the AlCl_3_-exposed rats. *Clostridium* and *Adlercruetzia* genera were evenly present in both groups before the *Lactobacillus* application, while on the 3rd and 10th day after *Lactobacillus* addition the ratio of *Adlercruetzia* genus decreased in both groups and the ratio of *Clostridium* genus decreased only in AlCl_3_-exposed rats. An abundant prevalence of *Lactobacillus* spp. was observed in the microbiota of the *Lactobacillus* treated rats even 10 days after the *Lactobacillus* application compared to the microbiota of the healthy and AlCl_3_-exposed rats. This increased abundance of the *Lactobacillus* genus possibly reflected an adaptation of *Lb. plantarum* SF9C and *Lb. brevis* SF9B in GIT as demonstrated by PCR-DGGE. However, besides allochthone lactobacilli SF9B and SF9C, PCR-DGGE indicated a presence of other commensal lactobacilli, suggesting the possible impact of the applied *Lactobacillus* strains on the competitive ability of autochthonous strains. The obtained results emphasised the influence of the applied *Lactobacillus* strains on rat microbiota composition, which will be valuable for further experiments on more experimental animals to investigate interactions of specific features of *Lactobacillus* strains, such as Slps or bacteriocin production, with the commensal members of gut microbiota. Further studies are needed to better understand the probiotic effects of these two strains on a healthy and disturbed gut microbiome composition and function, and the possible impacts on other parameters important in alleviating AlCl_3_-induced toxicity in host.

## Conclusion

The results of this research support an enhanced functionality potential of the joined application of *Lb. plantarum* SF9C and *Lb. brevis* SF9B strains in vivo. The cooperation between the two strains could result in a facilitated adhesion of *Lb. plantarum* SF9C due to the competitive pathogen exclusion by the coexisting *Lb. brevis* SF9B. Simultaneously, SF9B strain could benefit from the pathogen inhibition due to plantaricin production by SF9C strain, resulting in a broader spectrum of antibacterial activity. The plantaricin- and S-layer-expressing *Lactobacillus* strains could be promising probiotic candidates for combined application in functional food and for the treatment of different disorders linked with a dysbiosis of gut microbiota, which require further investigation.

## Materials and methods

### Bacterial strains, culture media and cultivation conditions

Table 3 shows bacterial strains and cultivation conditions used in this study.

**Table 3:** Bacterial strains used in this study

Bacterial strain	Cultivation conditions	References
*Lb. brevis* SF9B	MRS, 37 °C, microaerophilic	Banić et al. [6]
*Lb. plantarum* SF9C	MRS, 37 °C, microaerophilic	This study
*E. coli* 3014	BHI broth, 37 °C, aerobic	CIM-FFTB
*S.* Typhimurium FP1	BHI broth, 37 °C, aerobic	CIM-FFTB
*L. monocytogenes* ATCC^®^ 19111™	BHI broth, 37 °C, aerobic	ATCC
*S. aureus* 3048	BHI broth, 37 °C, aerobic	CIM-FFTB

CIM-FFTB—Culture collection of the Laboratory for Antibiotic, Enzyme, Probiotic and Starter Culture Technologies, Faculty of Food Technology and Biotechnology, University of Zagreb

*ATCC* American Type Culture Collection

Banić et al. [6] had already characterized S-layer-carrying *Lb. brevis* SF9B. Strains are deposited in the Culture Collection of the Laboratory for Antibiotic, Enzyme, Probiotic and Starter Culture Technologies, Faculty of Food Technology and Biotechnology, University of Zagreb (CIM-FFTB) and are maintained as frozen stock at -80 °C in appropriate medium supplemented with 15% (v/v) glycerol.

### Human cell line, culture medium and cultivation conditions

Enterocyte-like Caco-2 cells (Ruđer Bošković Institute, Zagreb, Croatia) were grown as monolayer cultures in RPMI 1640 medium (GIBCO, Carlsbad, CA, USA), supplemented with 15% fetal bovine serum (GIBCO, Carlsbad, CA, USA) and 4500 mg/L glucose. Cells were grown up to confluence at 37 °C and 5% CO_2_ in T-flasks, trypsinised and seeded into 24-multiwell plates. Prior to experiments, cells reached sub-confluence.

### DNA isolation and PCR analysis

Total genomic DNA, both for PCR analysis of the bacteriocin genes or WGS, was extracted according to the method of Leenhouts et al. [35] with minor modifications. The purity and concentration of the extracted DNA were then determined by using a BioSpec-Nano spectrophotometer (Shimadzu, Kyoto, Japan) and the extracted DNA was stored at − 20 °C. PCR screening of the bacteriocin structural genes was performed with primers listed in Table 4. Amplification of DNA fragments was performed in 50-µL reaction mixtures containing 25 µL Emerald Amp MAX HS PCR Mastermix Premix (TaKaRa, Ohtsu, Japan), 200 nmol/L each oligonucleotide primer, 300 ng DNA template and EmeraldAmp dH_2_O. A negative control, which contained all reagents except the DNA template, was used to detect contamination or non-specific amplification. The amplification was carried out in an Eppendorf Mastercycler personal thermal cycler (Eppendorf, Hamburg, Germany) using the conditions described by the authors cited in Table 4. PCR-amplified products were separated by electrophoresis in a 2% agarose gel, stained with ethidium bromide (0.5 μg/mL) and visualised on a MiniBIS Pro transilluminator (DNR Bio-Imaging Systems Ltd, Jerusalem, Israel) at 254 nm and images were captured by the GelCapture software v. 7.1 (DNR Bio-Imaging Systems Ltd, Jerusalem, Israel). The λ DNA *Hind*III (Fermentas, Waltham, MA, Canada) and 100 bp DNA Ladder (Invitrogen, Carlsbad, CA, USA) were used as molecular size standards.

**Table 4:** PCR primers used for the amplification of the plantaricin-related genes

Target gene	Bacteriocin	Forward primer (5′–3′)	Reverse primer (5′–3′)	Amplicon size (bp)	References
*plnA*	Plantaricin A	GTACAGTACTAATGGGAG	CTTACGCCAATCTATACG	450	Ben Omar et al. [9]
*plnEF*	Plantaricin EF	GGCATAGTTAAAATTCCCCCC	CAGGTTGCCGCAAAAAAAG	428	Ben Omar et al. [9]
*plnJ*	Plantaricin J	TAACGACGGATTGCTCTG	AATCAAGGAATTATCACATTAGTC	475	Ben Omar et al. [9]
*plnNC8*	Plantaricin NC8	GGTCTGCGTATAAGCATCGC	AAATTGAACATATGGGTGCTTTAAATTC	207	Maldonado et al. [37]
*plnS*	Plantaricin S	GCCTTACCAGCGTAATGCCC	CTGGTGATGCAATCGTTAGTTT	320	Ben Omar et al. [9]
*plnW*	Plantaricin W	TCACACGAAATATTCCA	GGCAAGCGTAAGAAATAAATGAG	165	Holo et al. [26]

### WGS and identification of genes encoding bacteriocins

Genomic DNA was prepared according to Frece et al. [20]. Genome sequencing was done using a paired-end approach as essentially described in Banić et al. [6]. Briefly, the Nextera DNA Library Preparation Kit (Illumina, San Diego, CA, USA) was used to construct a library. The library was processed with the Illumina cBot and sequenced on the MiSeq 2500 (Illumina, San Diego, CA,USA) pair-end with 300 cycles per read. Contigs were classified as belonging to *Lb. plantarum* when obtaining the best blastn v. 2.2.27 hit [2] in the NCBI nt database. RAST server, which identifies protein-encoding, rRNA and tRNA genes, assigns functions to the genes and predicts which subsystems are represented in the genome [5], was used for the annotation, distribution and categorization of all sequenced genes. The assembled contigs were compared with the so far identified bacteriocins in the NCBI database using the tblastn v. 2.2.27. To further supplement the annotation, BAGEL4 software was used to predict genes related to bacteriocin synthesis [46]. The input file was the genome sequence of *Lb. plantarum* SF9C in a fasta file. Conserved genes associated with the bacteriocin synthesis were retrieved using the RAST server [5]. Additionally, whole genome sequences were pairwise aligned with’run-mummer3‘to detect alignments and SNPs. The plot was computed with R package hclust, based on SNP frequency. SNP hierarchical clustering was created based on a high similarity of whole genome sequences available in the NCBI microbial genome database. The closest whole genome sequences of the 11 *Lactobacillus* strains in respect to the symmetrical and gapped identities among the draft and the complete genome sequences were selected for comparison. The references of the respected *Lactobacillus* strains isolated from different sources are as follows: WCFS1 [29], NC8 [4], RI-113 [28], B21 [23], BDGP2 [49], SF15C, SF9C, SF15B and SF9B [8], ATCC367 [36] and NCFM [1].

Furthermore, the 3D structure homology was modelled using the SWISS-MODEL server (https://swissmodel.expasy.org/) based on the alignment of the amino acid sequences of the core peptides, generated from BAGEL4 software. Additionally, helix properties of the plantaricins were calculated using heliQuest web server [22].

### In vitro assays

#### Testing of antimicrobial activity

The antimicrobial activity of the overnight grown culture of *Lb. plantarum* SF9C and *Lb. brevis* SF9B strains was tested against four different test microorganisms: *L. monocytogenes* ATCC^®^ 19111™, *S. aureus* 3048, *E. coli* 3014 and *S.* Typhimurium FP1 by agar spot test and well-diffusion method respectively. The agar spot test was performed according to Leboš Pavunc et al. [32]. The ratio of the inhibition diameter (ID) to the spot culture diameter (CD) was calculated to determine the effective inhibition ratio (EIR) of SF9C and SF9B strains: ((ID-CD)/CD). Furthermore, antimicrobial activity of the CFS of SF9C and SF9B strains was examined by the agar well-diffusion method, previously described by Kos et al. [31]. CFS was recovered by centrifugation, filtered through a 0.22-µm sterile filter (Millipore Corporation, Billerica, MA, USA) and concentrated up to fivefold in an Amicon cell concentrator (Amicon, Beverly, MA, USA) equipped with a selective (10 kDa) membrane. The proteinaceous nature of potential inhibitory compounds in CFS was examined by treatment with 1 mg/mL proteinase K (Invitrogen, Carlsbad, CA, USA) for 2 h at 37 °C and by heating the samples at 100 °C/30 min, according to Elayaraja et al. [18].

#### Evaluation of the antibacterial activity after cocultivation with the targeted pathogens

A slightly modified method of Kos et al. [31] served to determine the influence of cocultivation of *Lb. plantarum* SF9C with *L. monocytogenes* ATCC^®^ 19111™ and *S. aureus* 3048 on bacteriocin activity of SF9C strain. The number of viable cells was determined by spot-plate method using the corresponding selective media for each strain: MRS for lactobacilli, Baird-Parker (Oxoid, Hampshire, UK) for *S. aureus* and ChromoBio (Biolab Diagnostic Laboratory, Budapest, Hungary) for *L. monocytogenes* in 2-h intervals during the first 10 h, and after 22, 24 and 48 h of incubation. Plates were incubated for 24 h at 37 °C and the number of viable cells was expressed as log CFU/mL. Also, during the experiment, the antibacterial activity of SF9C strain in monoculture and coculture was tested by agar spot test as described above.

#### Pathogen competition and exclusion assay by *Lb. brevis* SF9B and *Lb. plantarum* SF9C on Caco-2 cell line

For exclusion and competition assay experiments, Caco-2 cells were routinely grown in 24-well culture plates until confluent differentiated monolayers were obtained. Cellular monolayers were carefully rinsed three times with PBS (pH = 7.4) before the addition of the bacterial cells. Two separate protocols were followed to assess the ability of viable strains of lactobacilli to inhibit *E. coli* 3014 and *S.* Typhimurium FP1 adhesion to Caco-2 cells. For both assays, *Lactobacillus* strains and pathogens were routinely cultivated; the cells were harvested and prepared in PBS (pH = 7.4) to reach *A*_620 nm_ = 1 (approximately 10^9^ CFU/mL). The competition assay was performed according to the procedure described by Uroic et al. [45] with some modifications. Lactobacilli and pathogens were co-incubated with Caco-2 monolayer for 1 h. For exclusion assays, *Lactobacillus* strains were cultured with Caco-2 monolayer for 1 h. Following a 1-h incubation, Caco-2 monolayers were gently washed three times with PBS (pH = 7.4), then pathogens were added and incubated for another 1 h. The 1.0-mL aliquots of the monospecies cultures of pathogenic bacteria together with 1.0 mL of EMEM per well were used as the controls in both assays. In all the above treatments, after the incubation, the non-adhered bacterial cells were removed by washing the Caco-2 monolayers three times with PBS (pH = 7.4). The Caco-2 cells were then lysed by the addition of 0.25% (v/v) Triton X-100 (AppliChem, Darmstadt, Germany) solution at 37 °C for 10 min in order to collect the adherent bacterial cells, and the total numbers of viable adhering *Lactobacillus*, *E. coli* and *S.* Typhimurium were determined by spot-plate method on MRS, Rapid (Biorad, Dubai, United Arab Emirates) and XLD (Biolife, Milano, Italy) agar plates, respectively. The efficiency of pathogen exclusion by *Lactobacillus* strains was assayed in three biologically independent experiments each with three replicates.

### In vivo animal trial

#### Preparation of *Lb. brevis* SF9B and *Lb. plantarum* SF9C strains and administration to rats

Bacterial cultures *Lb. brevis* SF9B and *Lb. plantarum* SF9C were grown in 5 mL of MRS broth at 37 °C under anaerobic conditions until the absorbance value reached 1.0 at 620 nm. Thus prepared cultures were mixed in 1:1 (v/v) ratio and inoculated (4%) in 50 mL of MRS broth. After overnight incubation under optimal conditions, the cells were harvested by centrifugation at 5000×*g* for 10 min, suspended in saline solution and the presence of both strains was microscopically examined. The bacterial suspensions were prepared daily to ensure viability and the CFU was controlled to maintain their constant number administered to a rat as it is described in the next chapter.

#### Experimental animals

Three-month-old male highly inbred Y59 strain rats, weighing 200 to 250 g (http://www.informatics.jax.org/external/festing/rat/docs/Y59.shtml), obtained from our breeding within the Department of Animal Physiology, Faculty of Science, University of Zagreb, were used in this study. The animals were maintained under a 12/12-h light–dark cycle with free access to food and water and standard housing conditions (room temperature around 25 °C and 60% humidity). They were fed a standard laboratory diet (4 RF 21, Mucedola, Settimo Milanese, Italy) and tap water ad libitum. Maintenance and care of all experimental animals were carried out according to the guidelines of the Republic of Croatia (Law on the Welfare of Animals, NN135/06 and NN37/13) and in accordance with EU Directive 2010/63/EU for animal experiments [40] and in compliance with the Guide for the Care and Use of Laboratory Animals, DHHS Publ. # (NIH) 86-123. The experimental procedure was approved by the Bioethics Committee of the Faculty of Science, University of Zagreb, Croatia (No. HR-POK-012).

#### Rat study design and sample collection

Male rats belonging to the Y59 inbred strain were randomly divided into 2 equally sized trial groups and housed three per cage in stainless-steel cages, under the same controlled conditions. The rats were treated daily for five consecutive days with a single dose (3 × 10^9^ CFU/mL) of *Lb. brevis* SF9B and *Lb. plantarum* SF9C strains suspended in saline solution, starting 24 h after the last treatment as follows: (a) first trial group represented a model of induced aluminium toxicity which was established by intraperitoneally injecting AlCl_3_ (10 mg/kg) and d-galactose (60 mg/kg) as described by Ulusoy et al. [44] and (b) second group served as healthy (control) group and was injected comparatively with saline solution in the same manner. No side effects were reported following *Lactobacillus* administration. In order to evaluate the AChE activity, which requires a brain sample, rats had to be sacrificed. Before the sacrifice, the rats were anaesthetized using a mixture of ketamine (75 mg/kg, Narketan^®^10, Vetoquinol AG, Belp Bern, Switzerland) with xylazine (10 mg/kg, Xylapana^®^ Vetoquinol Biowet Sp., Gorzow, R. Poland). The intestinal mucosal content from each sacrificed rat was scraped and specimens were kept frozen at − 80 °C until the analysis. The brain was removed and frozen at − 80 °C or kept in buffered formaldehyde until the analysis. The brain tissue homogenates were used to assess AChE activity by colorimetric method. AChE activity is expressed in mol/min/g tissue. The brain samples were prepared according to standard paraffin procedure. Changes related to early-stage Alzheimer’s disease were also (un)confirmed by immunohistochemistry using primary antibodies Purified-β-Amyloid, 17-24 Antibody (4G8) diluted 1:2000 (BioLegend, San Diego, CA, USA), Phospho-PHF-Tau (pSer202 + Thr205) Monoclonal Antibody (AT8) diluted 1:500 (Thermo Fisher Scientific, Waltham, MA, USA) and Iba1 diluted 1:250 (Wako Pure Chemical Industries, Osaka, Japan). Photomicrographs were recorded using a digital camera (AxioCam ERc5s, Zeiss, Germany) and processed by a computer program morphometric image analysis (AxioCam ERc5s-ZEN2). The faecal samples were collected from the cages before starting the treatment and on the 3rd and 10th day following the last *Lactobacillus* administration in triplicates, and stored at − 80 °C until analysis as described in the next chapter.

#### Bacterial 16S rRNA sequencing and processing using QIIME

Rat faecal samples were collected at the end of the study and the total genomic DNA was extracted using a Maxwell DNA Tissue Kit with automated extraction platform, Maxwell^®^ 16 Research System instrument (Promega, Madison, USA). The final equimolar pool was sequenced on the Illumina MiSeq platform using 341F (5′-CCTACGGGNGGCWGCAG-3′) and 518R (5′-ATTACCGCGGCTGCTGG-3′) primers. PCR reactions and 16S sequencing were performed at the Molecular Research LP (MRDNA, Shallowater, Texas, USA). The MiSeq instrument (Illumina) was used for sequencing the 16S amplicons following the manufacturer’s instructions at MRDNA described by Garcia-Mazcorro et al. [21] with slight modifications. Raw 16S data were obtained from Illumina’s basespace as FASTQ files and analysed with the QIIME 2 pipeline using the procedure as described in the ‘moving pictures’ tutorial (https://qiime2.org/).

#### PCR–DGGE analysis

PCR-DGGE analysis was performed according to Leboš Pavunc et al. [33] with slight modifications in order to check the presence of the *Lb. plantarum* SF9B and *Lb. brevis* SF9C in the faeces of *Lactobacillus*-fed rats. DNA was extracted directly from faecal samples of healthy rats for culture-independent PCR-DGGE analysis, as well as from the bacterial colonies, isolated on MRS agar plates for culture-dependent PCR-DGGE analysis, from faeces of healthy rats sampled before feeding (control), and on the 3rd and 10th day after the application of *Lactobacillus* SF9B and SF9C strains. In both cases, DNA was isolated using Maxwell DNA Cell Kit with automated extraction platform, Maxwell^®^ 16 Research System instrument (Promega, Madison, USA). The V2–V3 regions of the 16S ribosomal DNA gene of bacteria in the faeces contents or from pure cultures of lactobacilli were amplified with primers HDA1-GC (5′-ACTCCTACGGGAGGCAGCAGT-3′) and HDA2 (5′-GTATTACCGCGGCTGCTGGCAC-3′) [48]. To identify the lactobacilli recovered from rat faeces, the V2–V3 regions of the 16S rRNA gene of the strains were amplified. The amplicons were sequenced using ABI PRISM^®^ 3100-Avant Genetic Analyzer (Applied Biosystems, Foster City, CA, USA). A search of sequences deposited in the GenBank DNA database was conducted by using the BLAST algorithm. The identities of the isolates were determined based on the highest score.

#### Statistical analysis

All the experiments were repeated three times and the results were expressed as mean value of three independent trials ± standard deviation (SD). Statistical significance was appraised by one-way analysis of variance. Pairwise differences between the mean values of groups were determined by the Tukey’s honestly significant difference (HSD) test for post-analysis of variance pairwise comparisons (http://vassarstats.net). Statistical differences between groups were considered significant when *p* values were less than 0.05.

## Supplementary information

**Additional file 1: Fig. S1** Plantaricin-related genes of bacteriocinogenic strain *Lactobacillus plantarum* SF9C detected by PCR with a plantaricin structural gene-specific primers. S—standard (in bp).

**Additional file 2: Table S1.** Genes of *Lb. plantarum* SF9C involved in plantaricin production and their known or putative biochemical functions.

**Additional file 3: Fig. S2** Effective Inhibition Ratio (EIR) of test microorganisms, resulting from the antimicrobial activity of *Lb. plantarum* SF9C after the growth in coculture with: a) *S. aureus* 3048 (▓) and b) *L. monocytogenes* ATCC^®^ 19111™ (▓), and after the growth of SF9C alone (░), obtained by agar spot test. Each shown value is the mean ± SD. Asterisks indicate statistically significant differences of EIR of test microorganisms obtained by the *Lb. plantarum* SF9C after the growth in coculture with test microorganisms and alone, at the same incubation time: *p < 0.05, **p < 0.01.

**Additional file 4: Fig. S3** Photomicrograph of the sagittal section in a rat cerebellum; a control group (C) and AlCl_3_-exposed group. Morphological profile of the rat Purkinje cells (stained with Bielschowsky silver staining), diffuse plaques (4G8, scale bar 10 × = 100 µm) and expression of microglia cells markers Iba1 (scale bar 10 × = 100 µm; scale bar 40 × = 20 µm) in the molecular layer (ML) and granular layer (GL) of the cerebellum.

## Data Availability

The datasets used and/or analysed during the current study are available from the corresponding author on reasonable request.
