# Supplementary figures and images for: The functional capacity of plantaricin-producing Lactobacillus plantarum SF9C and S-layer-carrying Lactobacillus brevis SF9B to withstand gastrointestinal transit
Source: Microb Cell Fact. 2020 May 19;19:106. doi: 10.1186/s12934-020-01365-6 (PMC7236188; doi:10.1186/s12934-020-01365-6)

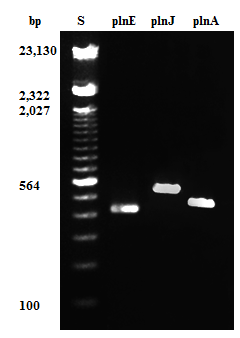

Supplement: Supplementary file 1 — Additional file 1: Fig. S1 Plantaricin-related genes of bacteriocinogenic strain Lactobacillus plantarum SF9C detected by PCR with a plantaricin structural gene-specific primers. S—standard (in bp). [file 12934_2020_1365_MOESM1_ESM.tif]

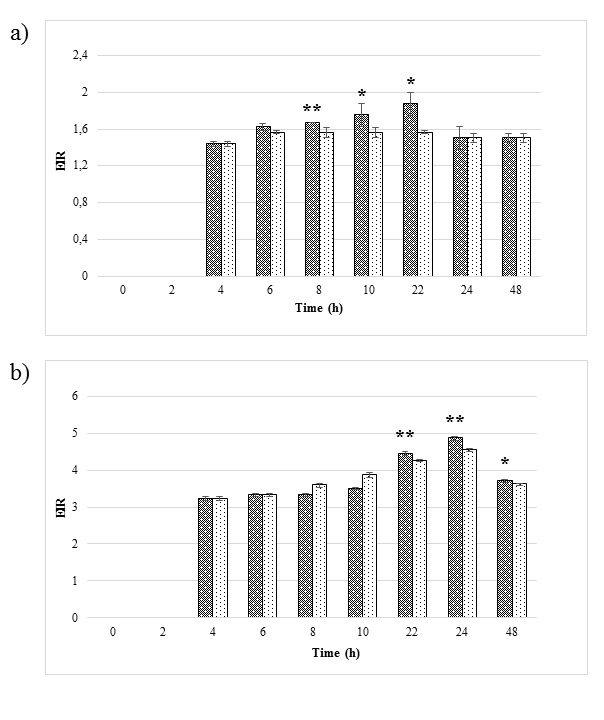

Supplement: Supplementary file 3 — Additional file 3: Fig. S2 Effective Inhibition Ratio (EIR) of test microorganisms, resulting from the antimicrobial activity of Lb. plantarum SF9C after the growth in coculture with: a) S. aureus 3048 (▓) and b) L. monocytogenes ATCC® 19111™ (▓), and after the growth of SF9C alone (░), obtained by agar spot test. Each shown value is the mean ± SD. Asterisks indicate statistically significant differences of EIR of test microorganisms obtained by the Lb. plantarum SF9C after the growth in coculture with test microorganisms and alone, at the same incubation time: *p < 0.05, **p < 0.01. [file 12934_2020_1365_MOESM3_ESM.tif]

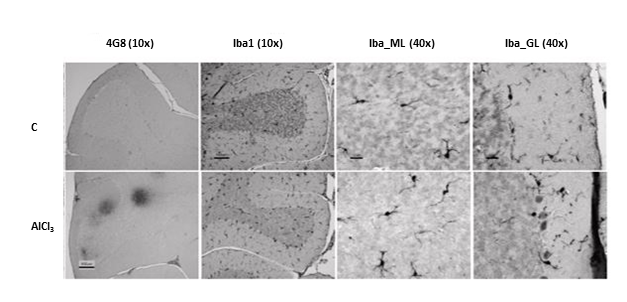

Supplement: Supplementary file 4 — Additional file 4: Fig. S3 Photomicrograph of the sagittal section in a rat cerebellum; a control group (C) and AlCl3-exposed group. Morphological profile of the rat Purkinje cells (stained with Bielschowsky silver staining), diffuse plaques (4G8, scale bar 10 × = 100 µm) and expression of microglia cells markers Iba1 (scale bar 10 × = 100 µm; scale bar 40 × = 20 µm) in the molecular layer (ML) and granular layer (GL) of the cerebellum. [file 12934_2020_1365_MOESM4_ESM.tif]
