# Supplementary material for: The functional capacity of plantaricin-producing Lactobacillus plantarum SF9C and S-layer-carrying Lactobacillus brevis SF9B to withstand gastrointestinal transit
Source: Microb Cell Fact. 2020 May 19;19:106. doi: 10.1186/s12934-020-01365-6 (PMC7236188; doi:10.1186/s12934-020-01365-6)
Supplement: Supplementary file 2 — Additional file 2: Table S1. Genes of Lb. plantarum SF9C involved in plantaricin production and their known or putative biochemical functions. [file 12934_2020_1365_MOESM2_ESM.docx]

**Additional file 2: Table S1.** Genes of *Lb. plantarum* SF9C involved in plantaricin production and their known or putative biochemical functions

| **Name** | **Perc. identity** | **e-value** | **Function** |
| --- | --- | --- | --- |
| **Genes encoding for the production of plantaricin** | | | |
| Plantaricin_K | 100.00% | 2e-37 | ComC; Bacteriocin_IIc; 173.2;Plantaricin_K |
| Plantaricin_J | 100.00% | 6e-37 | 172.2;Plantaricin_J |
| Plantaricin_N | 100.00% | 3e-35 | Bacteriocin_IIc; 174.2;Plantaricin_N |
| GlyS | 100.00% | 0.0 | PlnO |
| Plantaricin_A | 100.00% | 3e-29 | Antimicrobial17; Bacteriocin_IIc; 167.2;Plantaricin_A |
| orf00038 | 26.83% | 3e-17 | Bacteriocin production related histidine kinase |
| orf00039 | 99.55% | 7e-162 | Response regulator PlnC, activator |
| orf00040 | 99.19% | 7e-180 | response regulator PlnD |
| Plantaricin_F | 100.00% | 6e-35 | ggmotif; Lactococcin; Bacteriocin_IIc; 171.2;Plantaricin_F |
| Plantaricin_E | 100.00% | 7e-37 | 170.2;Plantaricin_E |
| **Genes involved in the synthesis of bacteriocin immunity proteins** | | | |
| orf00023 | 32.02% | 4e-20 | Putative bacteriocin Immunity protein |
| orf00029 | 100.00% | 2e-40 | P71462_LACPL Immunity protein PlnM |
| orf00032 | 29.86% | 5e-07 | P71468_LACPL PlnI (Immunity protein PlnI, membrane-bound protease CAAX family) |
| orf00044 | 99.61% | 6e-179 | P71468_LACPL PlnI (Immunity protein PlnI, membrane-bound protease CAAX family) |
| orf00058 | 31.75% | 3e-04 | PlnS |
| orf00059 | 38.89% | 3e-09 | PlnS |
| **Genes included in the transport of the produced plantaricin**  **outside the bacterial cell** | | | |
| LanT | 100.00% | 0.0 | Bacteriocin ABC-transporter, ATP-binding and permease protein PlnG |
| HlyD | 100.00% | 0.0 | Accessory factor for ABC-transporter PlnH |
| **Other genes** | | | |
| orf00001 |  |  | No function determined |
| orf00004 |  |  | No function determined |
| orf00005 | 58.25% | 3e-77 | Galactoside O-acetyltransferase |
| orf00006 | 23.57% | 2e-05 | Quinolone resistance protein NorA |
| orf00009 | 41.29% | 1e-63 | 5-amino-6-(5-phospho-D-ribitylamino)uracil phosphatase YbjI |
| orf00012 | 28.30% | 9e-16 | HTH-type transcriptional activator RhaR |
| orf00013 | 36.90% | 1e-49 | Sugar phosphatase YidA n=158 RepID=YIDA_ECOL6 |
| orf00015 | 41.24% | 2e-83 | Branched-chain amino acid transport system carrier protein |
| orf00018 | 50.13% | 8e-117 | Na(+)/H(+) antiporter |
| orf00020 |  |  | No function determined |
| orf00021 |  |  | No function determined |
| orf00034 |  |  | No function determined |
| orf00055 | 35.78% | 1e-05 | Putative membrane peptidase YdiL |
| orf00056 |  |  | No function determined |
| orf00060 |  |  | No function determined |
| orf00062 | 34.41% | 8e-150 | DNA helicase IV |
